# The Oncogenic Response to MiR-335 Is Associated with Cell Surface Expression of Membrane-Type 1 Matrix Metalloproteinase (MT1-MMP) Activity

**DOI:** 10.1371/journal.pone.0132026

**Published:** 2015-07-23

**Authors:** Fausto Rojas, Maria E. Hernandez, Milagros Silva, Lihua Li, Subbaya Subramanian, Michael J. Wilson, Ping Liu

**Affiliations:** 1 Centro de Investigaciones Cerebrales, Universidad Veracruzana, Xalapa, Veracruz, Mexico; 2 Department of Laboratory Medicine and Pathology, University of Minnesota, Minneapolis, Minnesota, United States of America; 3 Department of Surgery, University of Minnesota, Minneapolis, Minnesota, United States of America; 4 Masonic Cancer Center, University of Minnesota, Minneapolis, Minnesota, United States of America; 5 Department of Pharmacology, University of Minnesota, Minneapolis, Minnesota, United States of America; 6 Minneapolis VA Medical Center, Minneapolis, Minnesota, United States of America; Stony Brook University, UNITED STATES

## Abstract

MicroRNA miR-335 has been reported to have both tumor suppressor and oncogenic activities. In order to determine possible tissue and cell type differences in response to miR-335, we examined the effect of miR-335 on cell expression of MT1-MMP, a proteinase commonly expressed in tumors and associated with cell proliferation and migration. miR-335 increased cell surface expression of MT1-MMP in fibrosarcoma HT-1080 and benign prostate BPH-1 cells, but not in prostate LNCaP or breast MCF-7 tumor cells. miR-335 stimulated proliferation and cell migration in a wound healing *in vitro* assay in HT-1080, BPH-1, and U87 glioblastoma cells, cells which demonstrated significant cell surface expression of MT1-MMP. In contrast, miR-335 did not affect proliferation or migration in cells without a prominent plasma membrane associated MT1-MMP activity. Our data suggest that differences in response to miR-335 by tumor cells may lie in part in the mechanism of regulation of MT1-MMP production.

## Introduction

MicroRNAs (miRNAs) are a class of small (~21 nucleotides) noncoding RNAs that regulate important cellular pathways of diverse normal biological processes including cell proliferation, differentiation, motility, development and apoptosis, as well as pathologies such as cancer. They negatively regulate gene expression by binding to 3’-untranslated regions (3’-UTRs) of specific mRNAs and block their translation or promote their destruction. Each miRNA can regulate multiple target genes and each mRNA in turn can contain target sites that interact with other miRNAs. It is estimated that approximately one third of all mammalian protein-coding genes are directly regulated by miRNAs [1]. In this manner, miRNAs can potentially function in cancer as oncogenes or tumor suppressors, depending on the function of the proteins and their levels being regulated. In this regard, miRNAs have been found to promote (e.g., miR-106, miR-373, miR-520c) and suppress (e.g., miR-335, miR-31, miR-206, miR-146a/b) specific steps in metastatic pathways. miR-335 is considered a tumor suppressor as it was found to be down-regulated in breast cancer [2–4], an effect resulting in part from genetic deletion of miR-335 and hyper-methylation of its promoter [5]. Over expression of miR-335 in breast cancer cells suppressed migration, invasion and metastatic colonization without inhibiting proliferation [2]. Additional studies of this miRNA found it to be down-regulated in clear cell renal cancer [6], pediatric acute lymphoblastic leukemia [7], non-small cell lung cancer [8], and in differentiation of mesenchymal stem cells [1]. However, other studies of miR-335 have found it to be elevated in multiple myeloma [9], meningiomas [10], human glioma [11], colorectal cancer [12, 13], and malignant astrocytomas [14]. In contrast to the breast cancer studies above, over expression of miR-335 was determined in tissues of that cancer [15], and both up- and down-regulation of miR-335 have been reported for gastric cancer [16, 17].

There is substantial evidence for a causal role of matrix metalloproteinases (MMPs), especially membrane-type 1 MMP (MT1-MMP, MMP-14), in mediating pericellular proteolysis of a large array of proteins that regulate cell properties such as adhesion, proliferation, and motility, which in turn enable tumor cells to become invasive and metastatic [18–25]. MT1-MMP has been implicated in the aggressiveness of a variety of cancers and the cell surface activation of proMMP-2 and proMMP-13 facilitates MT1-MMP in this role. The expression and function of MT1-MMP are controlled at multiple levels including transcription, translation, activation of the pro-enzyme by pro-protein convertases, inhibition by specific inhibitor proteins (TIMPS and RECK), and trafficking to and from the cell surface [21–23, 26, 27]. In view of the divergent reports indicating miR-335 can have tumor suppressor or promoter roles in different tumors, we proposed to study the cell surface expression of MT1-MMP, a tumor cell property central to tumor growth, invasion and metastasis. Our study indicates that miR-335 can regulate cell surface MT1-MMP levels in some tumor cells, a property accompanied by increased motility and proliferation in these cells.

## Materials and Methods

### Cell culture, treatment conditions, and transfection

Human fibrosarcoma cell line HT1080, human breast cancer cell lines MCF7 and MDA-MB-231, and human primary glioblastoma cell line U87 were from ATCC (Monassas, VA); colon cancer cell line HCT116 (originally from ATCC, Manassas, VA) and the immortalized human benign prostate hyperplasia epithelial cell line BPH-1 [28] were kindly provided by Dr. Clifford Steer and Dr. Haojie Huang, University of Minnesota, respectively. HCT116 and BPH-1 cells were routinely cultured in RPMI-1640 media and HT1080, U87, MCF7, and MDA-MB231 cells using DMEM media. Both media were supplemented with 10% heat-inactivated FBS and 1% (V/V) penicillin-streptomycin (10,000 U/ml penicillin and 10 mg/ml streptomycin in 0.9% NaCl). All cells were cultured within a growth chamber with 5% CO_2_ and 95% air at 37°C. Upon reaching 60–70% confluence, the cultures were changed to serum-free medium or media with 5% heat inactivated FBS and appropriate treatment agents and were continuously cultured for 60 hr [48 h for Concanavalin A (ConA)experiments] at which time culture media, cell protein extracts, or cellular RNA were isolated. Treatments included 0, 10 or 50 μg/ ml ConA (Sigma Chemical Co., St. Louis, MO). Media were centrifuged at 12,000 rpm for 10 min, and frozen and stored at -80°C until used. Cell protein extracts were prepared by homogenizing cells with 100 μl SoluLyse M Mammalian Protein Extraction Reagent proprietary nonionic detergent (cat L-30012, Genlantis Inc., San Diego, CA) in 25 mM Tris pH 7.4 and 250 mM sucrose and containing a mixture of protease inhibitors (cat No. 11836153001, Roche Diagnostics GmbH, Mannheim, Germany). miR-335 mimic and miR-control were purchased from Ambion (Foster City, CA) and were transfected into cells using Lipofectamine 2000 in accordance with the manufacturer’s procedure (Invitrogen, Carlsbad, CA).

### Gelatin zymography

Zymographic gel assays of culture media were performed as described previously [29]. Equal amounts of serum-containing media (5 μl) were subjected to electrophoresis in triplicate 0.1% gelatin-8.5% polyacrylamide gels in the presence of sodium dodecyl sulfate (SDS) under non-reducing conditions. After electrophoresis, gels were rinsed with distilled water, washed with 2.5% Triton X-100 solution at room temperature with gentle agitation (2Xs for 15 min) to remove SDS, and incubated in reaction buffer (50 mM Tris-HCl pH 8.4, 5 mM CaCl_2_, 10 μM ZnCl_2_ and 0.02% NaN_3_) at 37°C overnight (16–18 h). The gels were subsequently stained with Coomassie blue, destained, and gelatinolytic activity of MMPs was visualized as a clear band against a dark background of stained gelatin.

### Western blotting

After culturing transfected cells 60 h, cells were harvested and protein extracts prepared and analyzed by western blotting [29, 30] to assess the level of proteins expressed. Proteins (50 μg) were electrophoresed in 12% polyacrylamide gels in the presence of SDS and reducing conditions and blotted onto PVDF membranes. Antibodies used were anti-human MT1-MMP (sc-30074, 1:500, Santa Cruz Biotechnology, Dallas, TX), and anti-human GAPDH (sc-25778,1:500, Santa Cruz Biotechnology) followed by a goat anti-rabbit IgG-HRP (sc-2004, 1:4,000, Santa Cruz Biotechnology). The bands were detected using the ECL Plus Western Blotting Detection Reagents (Amersham, Little Chalfont, Buckinghamshire, UK) according to specifications. Quantification of western blots was done by using Adobe Photoshop C53 Extended to measure the gray / intensity on a black/white inversion of the blot.

### RNA extraction and quantitative real time-PCR (qPCR)

Total RNA and microRNAs were isolated from cells using the miRvana miRNA isolation kit following the manufacturer’s protocol (Ambion, Applied Biosystems, Grand Island, NY). cDNA synthesis was performed at 37°C for 60 min and 95°C for 5 min by using a miScript Reverse transcription kit (Qiagen, Valencia, CA). EMMPRIN and MT1-MMP mRNA and miR-335 were examined in a LightCycler 480 (Roche Applied Science, Indianapolis, IN) by using QuantiTect SYBR Green PCR kit according to the manufacturer’s protocol (Qiagen). GAPDH and U6 were used as internal controls to check the efficiency of cDNA synthesis and PCR amplification [31]. The PCR primers were MT1-MMP: (forward) 5’ GGGTCTCAAATGGCAACATAATGA 3’ and (reverse) 5’ ATGGAAGCCCTCGGCAAA 3’; EMMPRIN (CD147): (forward) 5’ CCATGCTGGTCTGCAAGTCAG 3’ and (reverse) 5’ CGTTGCACCGGTACTCGC 3’.

### Wound healing/scratch test

In brief, cells were seeded in 12 well plates. After growth to about 70–80% confluency, cells were transfected with the miR-335 mimic or control miR, and then grown to confluency. Cell motility was then tested using a wound healing assay in which a scratch was made along the axis of the plate using a 10 μl pipette tip. Cells were washed two times with PBS buffer to remove free cells, complete media with 5% FBS was added, and cells were grown under normal conditions. Migration of cells into the scratch was photographed every 4 h. Quantification was carried out with Sigma Scan Pro version 5.0 software by measuring the remaining area of the wound.

### Cell proliferation assay

Cell proliferation was measured using the Cell Counting Kit-8 (CCK-8) of Dojindo Molecular Technologies, Inc. (Rockville, MD) following the manufacture’s protocol. In the assay the tetrazolium salt WST-8 is reduced by dehydrogenase activities of the cells to give a yellow color formazan dye, which is proportional to the number of living cells.

### Immunostaining and confocal microscopy

Confocal microscopy was carried out as described previously [30]. Cells were grown on glass cover slips and treated as indicated. After culture for 48 hr in 10% FBS media, cells were fixed with 4% paraformaldhyde, incubated with 0.1% Triton X-100 in PBS, blocked with 3% goat serum in PBS, and then incubated with mouse anti-human MT1-MMP antibody (Santa Cruz Biotechnology) overnight at 4°C. The secondary antibody was Alexa Fluor488-labeled goat anti-mouse IgG (Invitrogen, Carlsbad, CA). Confocal microscopy was carried out in the University of Minnesota Imaging Center using an Olympus FluorView 1000 BX2 Upright Confocal Microscope with a 60X oil objective. The images were processed in Power Point 14.3.1 software for Mac.

### Data analysis and statistics

Experiments were carried out with three replicates. All quantitative data were presented as mean +/- S.D. Statistical analysis was done with ordinary one-way ANOVA for Quantitative Real Time PCR and two-way ANOVA for cell proliferation assay using the Prism 6 for Mac OSX computer program. Values of p<0.05 were considered significant.

## Results

**Fig 1:**
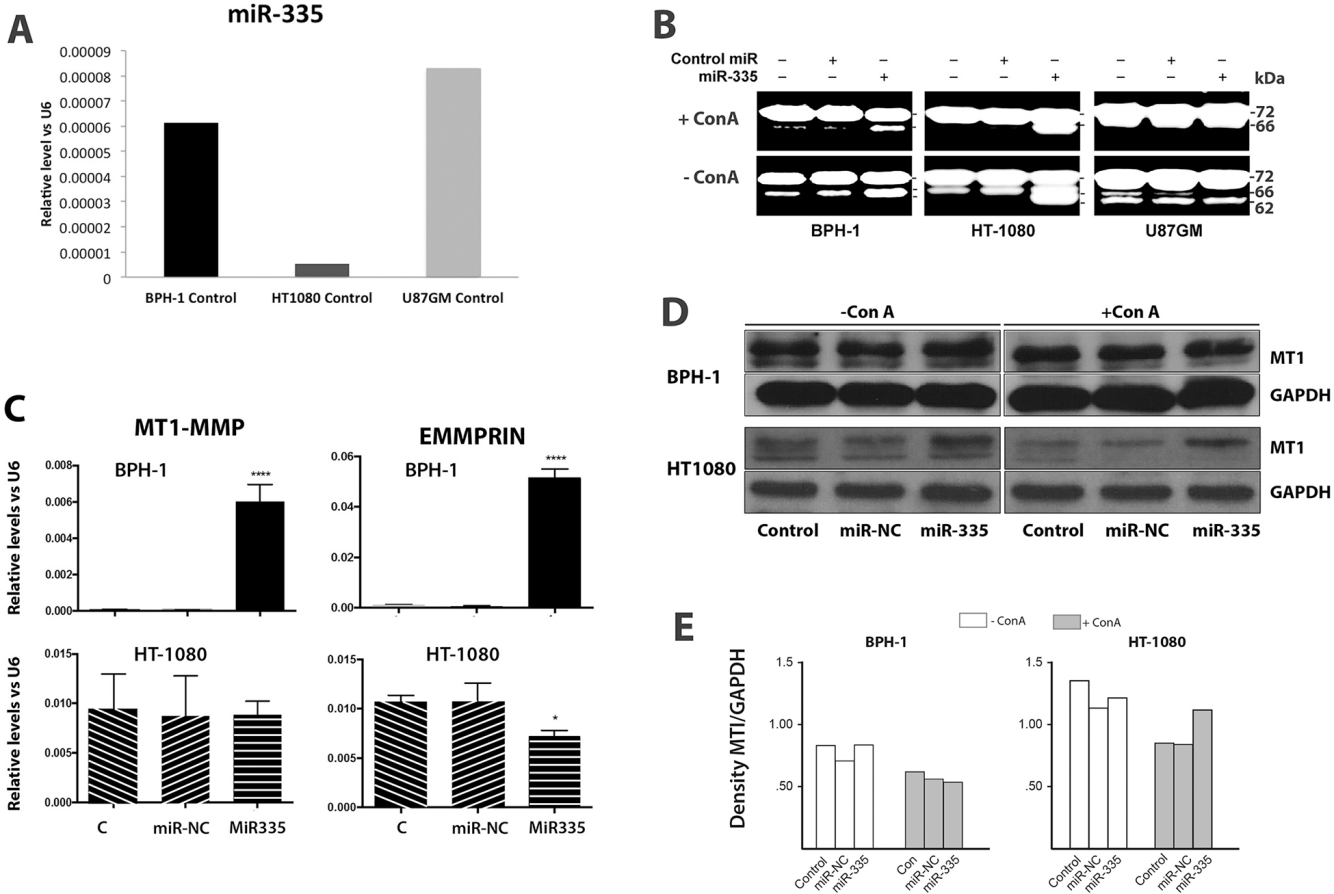
The effect of miR-335 on MT1-MMP expression and cell surface activation of proMMP-2. (A) The expression of miR-335 in untreated HT-1080, BPH-1 and U87GM cells as measured by real-time RT-PCR. The base-line level of miR-335 was about 15 and 25 fold greater in BPH-1 and U87GM cells respectively, than in HT-1080 cells. (B) miR-335 stimulation of cell surface MT1-MMP activity. The addition of miR-335 in contrast to a control miR (miRNC) or no addition stimulated proMMP-2 activation in HT-1080 and BPH-1 cells, but not U87GM cells. The addition of ConA increased proMMP-2 activation in all three cell lines, and miR-335 stimulated proMMP-2 activation in HT-1080 and BPH-1, but not U87GM cells. (C). The effect of miR-335 on expression of mRNA for MT1-MMP and EMMPRIN in relation to control no addition or control miRNA (miR-NC) as determined by real-time RT-PCR in BPH-1 and HT-1080 cells. The messages for both MT1-MMP and EMMPRIN were increased by miR-335 in BPH-1 cells but were unchanged (MT1-MMP) or decreased (EMMPRIN) by miR-335 in HT-1080 cells. (D and E) The effect of miR-335 on the protein levels of MT1-MMP in BPH-1 and HT-1080 cells determined by immuno-blotting (D) and quantification of the level of MT1-MMP as the ratio of MT1-MMP/GAPDH (E). The level of MT1-MMP was increased in HT-1080 cells treated with miR-335 alone or with addition of ConA. No change in the level of MT1-MMP in BPH-1 cells was evident.

The role of miR-335 on possible regulation of MT1-MMP expression was undertaken using qPCR in HT-1080 fibrosarcoma and U87GM glioblastoma tumor cells, and in BPH-1 nonmalignant prostate cells. The endogenous levels of miR-335 in untreated HT-1080 cells was substantially lower than either BPH-1 or U87GM cells, by about 15 and 25 fold respectively (Fig 1A).

The effect of miR-335 on cell surface MT1-MMP function was tested by examining the ability of cells to activate proMMP-2 present in the serum of the cell culture media (Fig 1B). miR-335 increased proMMP-2 processing in the human fibrosarcoma cell line HT-1080 and in the benign prostatic hyperplasia cell line BPH-1, but not the glioblastoma cell line U87-GM. The effect of miR-335 on proMMP-2 processing was enhanced in HT-1080 and BPH-1 cells by ConA, and although ConA stimulated proMMP-2 activation in U87GM cells, there was no further effect of miR-335 on this process. MCF7 and MDA-MB-231 breast cancer cell lines did not demonstrate proMMP-2 activation even with ConA, indicating the absence of MT1-MMP on the cell surface. These cells also showed no response to miR-335 on cell surface activation of proMMP-2 (data not shown). Pro-MMP-9 was secreted by BPH-1 and HT-1080 cells into the media, but the amount expressed was not increased with ConA, miR-335, or combination of ConA and miR-335 treatment. In addition, there was no activation of pro-MMP-9 detected in the zymographic gels without or with addition of miR-335 (data not shown).

**Fig 2:**
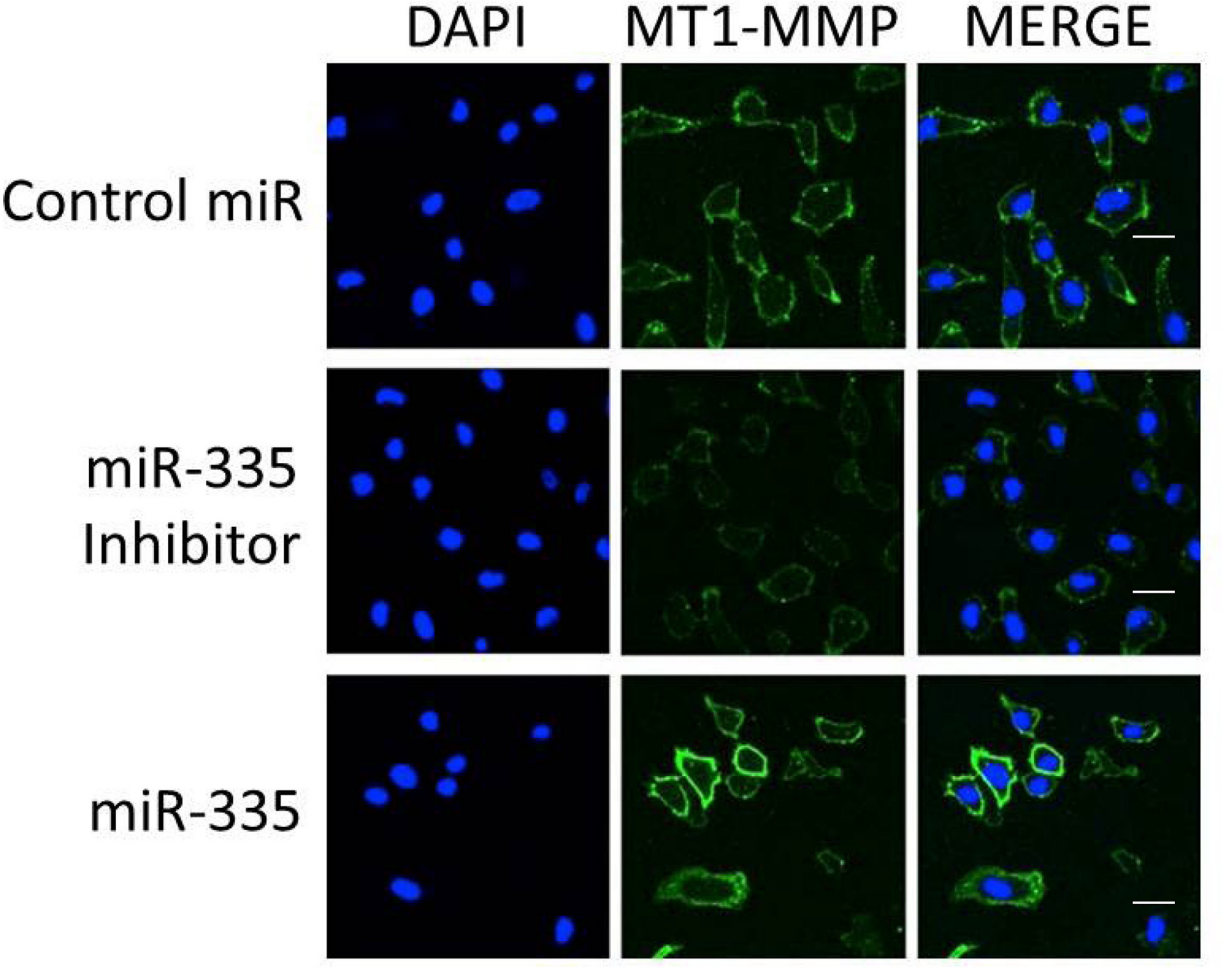
The effect of miR-335 on cell surface localization of MT1-MMP as determined by confocal microscopy in HT-1080 cells. The level of cell surface MT1-MMP observed in control miR treated cells was reduced by treatment with a miR-335 inhibitor. In contrast, cell surface localization of MT1-MMP was increased by miR-335. The bars indicate 20 microns.

The levels of MT1-MMP transcript were measured by qPCR in BPH-1 and HT-1080 cells and were found to be increased by treatment with miR-335 in the former but not the latter (Fig 1C). The level of MT1-MMP transcript was increased by miR-335 as compared to a control miRNA or sham treated cells. In addition, the levels of transcripts for EMMPRIN, an inducer of MMP expression [18], were found to be increased in BPH-1, but relatively decreased in HT-1080 cells. The protein levels of MT1-MMP were not changed in BPH-1 cells treated with miR-335 alone or together with ConA (Fig 1D and 1E) but demonstrated a small elevation in miR-335 stimulated HT-1080 cells treated with ConA (Fig 1D and 1E). The distribution of MT1-MMP in HT-1080 cells was also examined by confocal microscopy (Fig 2). Treatment with miR-335 increased the cell surface localization of MT1-MMP as compared with cells transfected with the control miRNA sequence. This effect supports the increased level of proMMP-2 activation found in Fig 1B. Blocking the effect of miR-335 by using an inhibitor decreased the level of MT1-MMP expression, as well as surface localization.

**Fig 3:**
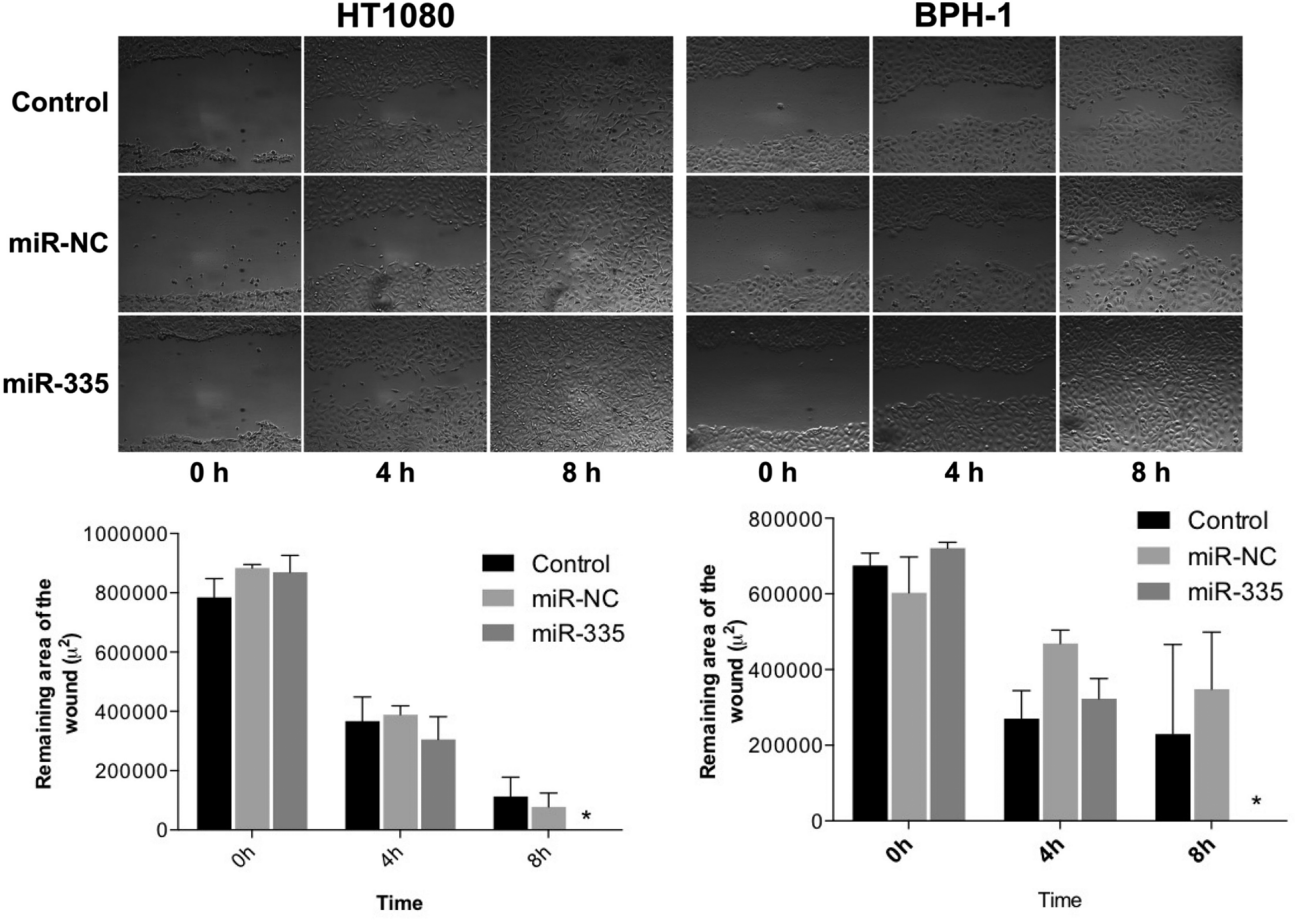
The effect of miR-335 on cell migration of HT-1080 and BPH-1 cells in an in vitro model of wound healing. Treatment of cells with miR-335 increased the rate of cell migration observed at 4 and 8 hours after a scratch was made in a confluent layer of cells using a pipette tip. The data were quantified as Remaining Area of Wound (μ^2^).

**Fig 4:**
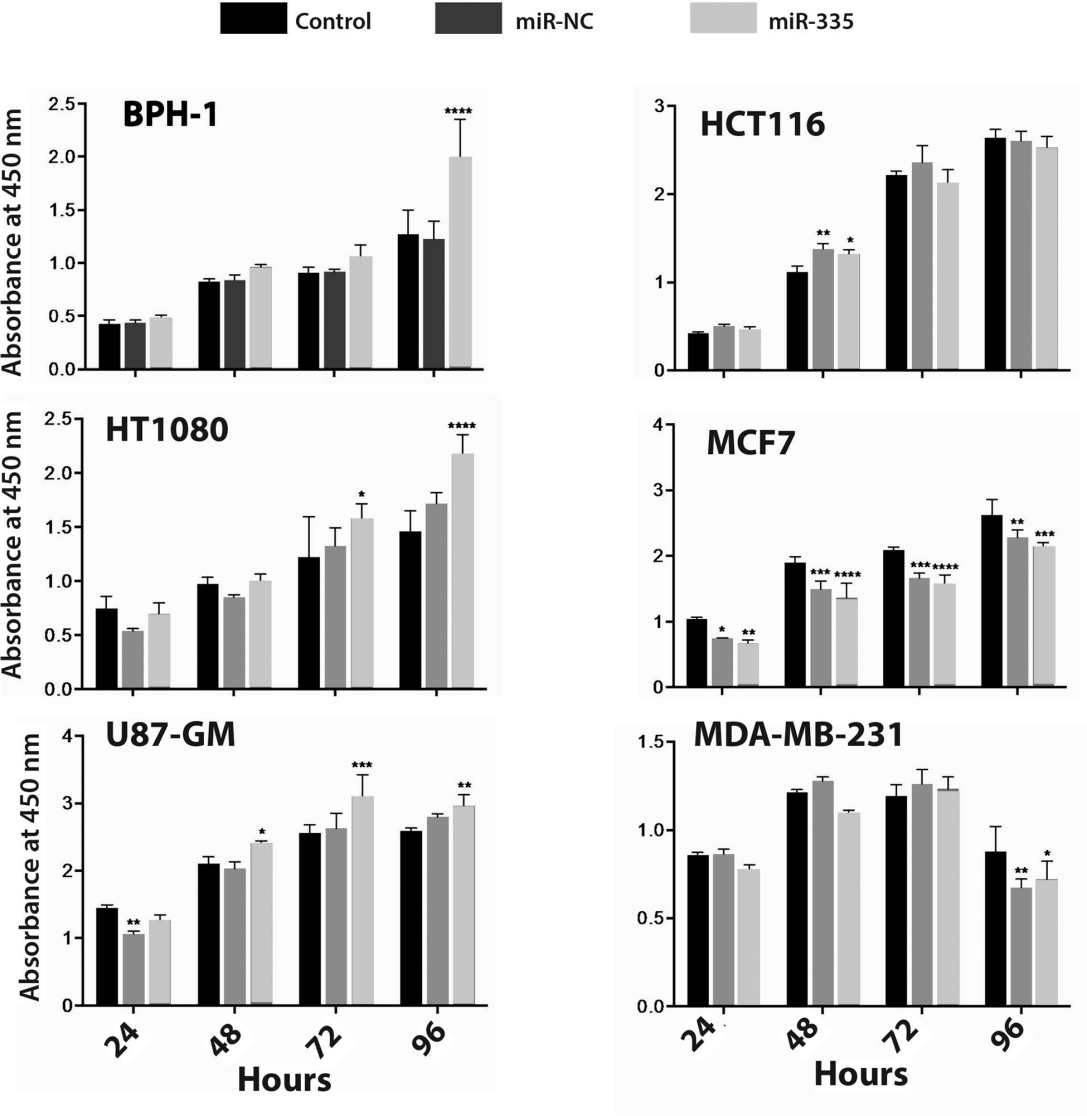
The effect of miR-335 on cell proliferation. Cell proliferation was measured using the CCK-8 assay. Increased levels of proliferation in miR-335 treated cells were found at 72 and 96 hrs for HT-1080, 96 hrs for BPH-1, and 72 hrs for U87GM cells. There was no change in rate of proliferation in HCT116, and a decrease in proliferation of MCF7 and MDA-MB-231 cells.

Having found miR-335 to affect differences in cell surface localization of MT1-MMP, we examined the effect of miR-335 on cell migration and proliferation. Both HT-1080 and BPH-1 cells transfected with miR-335 showed increased motility compared to cells untreated or treated with the control miR in an *in vitro* (scratch test) wound healing assay (Fig 3). In contrast, U87GM cells, and MCF7 and MDA-MB-23 breast cancer cells did not respond to miR-335 in change of rate of filling the void with cells (data not shown). HT-1080 and BPH-1 cells responded to miR-335 with increased cell proliferation, HT-1080 at 72 and 96 hours post-treatment, and BPH-1 cells at 96 hours (Fig 4). U87GM cells demonstrated a small enhancement of cell proliferation at 72 hrs, whereas HCT116 cells did not change their rate of proliferation and MCF7 and MDA-MB-231 cells showed a decreased rate of proliferation in response to miR-335. The response to miR-335 resulting in increased cell migration and proliferation occurred in those cell lines that responded to the microRNA with increased cell surface localization of MT1-MMP.

## Discussion

Our studies have shown that tumor cell lines that respond to miR-335 with increased cell surface MT1-MMP also demonstrated increased cell proliferation and migration. BPH-1cells also had increased expression of EMMPRIN, a protein which stimulates MMP production [18]. Thus, the role of miR-335 in up-regulating MT1-MMP expression may lie upstream of MT1-MMP by blocking synthesis of protein transcription factors that prevent up-regulation of the MT1-MMP gene. This proposed mechanism of MT1-MMP expression regulation in our study is evident for miR-106 which targets HOXD10, a transcriptional repressor that inhibits several genes involved in cell migration and extracellular matrix remodeling, including MT1-MMP and uPAR [32–34]. In contrast, miRNAs can also block expression of transcription factors that positively regulate MT1-MMP expression. miR-145 down regulates HIF-2α by targeting its 3’-UTR, and by decreasing the level of this transcription factor, repressed expression of downstream genes cyclin D1, VEGF, and MT1-MMP, and via this means, tumor cell growth, invasion, and metastasis [35]. miRNAs can also negatively regulate MT1-MMP expression by directly blocking MT1-MMP mRNA translation. In this light, decreased MT1-MMP expression by miR-9, miR-133a, and miR-24 is achieved by directly targeting putative binding sites in the 3’-UTR of MT1-MMP mRNA [36–38]. The differences in tumor cell MT1-MMP expression response to miR-335 may lie in differences in tissue specificity reflected in predominance of certain cellular regulatory pathways and the overall balance of genes targeted by miR-335. Utilizing bioinformatics data, Yan et al. [16] determined 255 genes were predicted as targeted by miR-335 and many of these were in the same pathways such as p53, MAPK, TGFβ, Wnt, ERbB, mTOR, and focal adhesion. MT1-MMP was not indicated as a target, nor did we find a binding site in the 3’-UTR of MT1-MMP in the NCBI Database utilizing TargetScanHuman (Version 6.2). Our data suggest that the differences in response to miR-335 by tumor cells may lie in part in the mechanism of regulation of MT1-MMP production.

The HT-1080 and BPH-1 cells which responded to miR-335 with increased cell surface MT1-MMP localization, and U-87 cells, which did not respond to miR-335 but already had a strong presence of MT1-MMP on the cell surface, were stimulated to proliferate by miR-335. U87 cells, which had a higher endogenous level of miR-335 than HT-1080 and BPH-1 cells, have a significant amplification of the miR-335 locus [14] and may have sufficient endogenous miR-335 so that an additional MT1-MMP response to miR-335 is not observed. Those cell lines with little or no cell surface MT1-MMP activity did not respond to miR-335 with proliferation. miR-335 appears to control growth by blocking cell proliferation by targeting certain genes; e.g., RASA1 in rat epididymal development [39], SP1 and Bcl-w in non-small cell lung cancer [8], SOX4 and TNC in breast cancer [2], ROCK1, MAPK1, and LRG1 in neuroblastoma [40], and Rb1 in U2OS osteosarcoma cells [41]. Thus, decreasing levels of miR-335 rescues cells from their negative regulatory mechanisms resulting in increased growth and invasive character of the tumor. On the other hand, miR-335 can target genes that suppress cell proliferation. Cell proliferation is increased in U87 cells via Wnt/PCP signaling by miR-335 decreasing DAAM expression [14] and miR-335 targets RB1 in meningioma cells resulting in increased cell growth and inhibited cell cycle arrest at the GO/G1 transition [10]. Up-regulation of miR-335 in glioma cells reduces expression of PAX6, thus promoting cell proliferation and is accompanied by increased protein levels of MMP-2 and MMP-9 [42]. These data indicate miR-335 has a complex role in tumorigenesis and its regulatory function may be tissue specific.

MT1-MMP promotes cell proliferation in several ways. It can affect the cell microenvironment by cleaving type I collagen in 3 dimensional (3 D) gels to produce areas for cell growth [21, 22], by destructing or impeding formation of 3D fibronectin matrices enclosing cells [43], by shedding of growth factors such as TGFβ from the extracellular matrix [44], or cleaving cell surface proteins such as notch 1 in melanoma cells [45]. Studies have also indicated that MT1-MMP may stimulate cell proliferation and migration (independent of its proteolytic activity) by activating the Ras-Raf-ERK signaling cascade through binding TIMP-2 [22, 46] and by functions not at the cell surface; i.e., localizing to centrosomes of dividing cells (including cells with low levels of MT1-MMP expression) where it can cleave pericentrin-2 [47]. Gene expression profiling has identified a number of target downstream genes linked transcriptionally to MT1-MMP expression; these genes include regulators of a number of cellular functions fundamental to cell proliferation and migration [48].

The same cells stimulated to proliferate by miR-335 also responded to the miRNA in filling the void of cells created in the *in vitro* wound healing assay, and those cells with little/no cell surface MT1-MMP did not. A considerable amount of MT1-MMP in many cells is intracellular, whereas, cell surface MT1-MMP expression is usually weak and may be in a complex with TIMP-2 [21, 49]. Control of cell surface localization and distribution of MT1-MMP involves its cytoplasmic domain and is regulated temporally and spatially by interaction with plasma membrane proteins such as the tetraspanins [21, 22]. A role for MT1-MMP in cell migration and invasive behavior has been supported by numerous studies. MT1-MMP mediates invasion of hydrogels with pore sizes of 1–2 μm in diameter in which proteolysis results in matrix pore size sufficient to permit protease-independent cell migration [50]. Cell locomotion may be mediated by an amoeboid motility utilizing elevated actomyosin contractility allowing cells to extend bleb-like protrusions or in part by invadopodia which are specialized actin-rich adhesion protrusions of cells in which MT1-MMP is the primary protease component. MT1-MMP is transported from the Golgi via endosomes to the cell surface, and invadopodia, in response to extracellular stimuli via ligand activating mitogenic receptor tyrosine kinases and subsequently internalized via the endo/exocytic pathway to lysosomes for degradation or to be recycled back to the cell surface [49]. In addition, cleavage of extracellular matrix proteins such as type I collagen (a well-established substrate of MT1-MMP [18, 22, 23]) and cellular proteins such as syndecan-1, CD44, and laminin-322 by MT1-MMP speeds migration of cells in culture and keratinocyte outgrowth from skin explants in vitro is reduced in MT1-MMP^-/-^ mice [51].

In conclusion, the data presented here suggest that a distinction in cell types that respond to miR-335 by increased cell proliferation and migration is related to their ability to place MT1-MMP on the cell surface. The functional role of miR-335 in the up-regulation of MT1-MMP remains to be established but would appear to involve down-regulation of gene products that would negatively regulate MT1-MMP expression, or expression of a protein like EMMPRIN, which in turn stimulates MT1-MMP production.

## References

[1] Tome’ M, Lopez-Romero P, Albo C, Sepulveda JC, Fernandez-Gutierrez B, Dopazo A, et al, miR-335 orchestrates cell proliferation, migration and differentiation in human mesenchymal stem cells. Cell Death Diff 2011;18: 985–995. 10.1038/cdd.2010.167 PMC3131940 21164520

[2] Travazole SF, Alarcon C, Oskarsson T, Padua D, Wang Q, Bos PD, et al, Endogenous human microRNAs that suppress breast cancer metastasis. Nature 2008;451: 147–152. 10.1038/nature06487 18185580 PMC2782491

[3] Heyn H, Engelmann M, Schreek S, Ahrens P, Lehmann U, Kreip H, et al, MicroRNA miR-335 is crucial for the BRCA1 regulatory cascade in breast cancer development. Int J Cancer 2011;129: 2797–2806. 10.1002/ijc.25962 21618216

[4] Wang F, Zheng Z, Guo J, Ding, Correlation and quantitation of microRNA aberrant expression in tissues and sera from patients with breast tumor. Gynecol Oncol 2010;119: 586–593. 10.1016/j.ygyno.2010.07.021 20801493

[5] Png KJ, Yoshida M, Zhang XH-F, Shu W, Lee H, Rimner A, et al, MicroRNA-335 inhibits tumor reinitiation and is silenced through genetic and epigenetic mechanisms in human breast cancer. Genes & Devel 2011;25: 226–231. 21289068 10.1101/gad.1974211 PMC3034897

[6] White NM, Bao TT, Grigull J, Youssef YM, Girgis A, Diamandis M, et al, miRNA profiling for clear cell renal carcinoma: biomarker discovery and identification of potential controls and consequences of miRNA dysregulation. J Urol 2011;186: 1077–1083. 10.1016/j.juro.2011.04.110 21784468

[7] Yan J, Jiang N, Huang G, Tay JL, Lin B, Bi C, Koh GS, et al, Deregulated miR335 that targets MAPK1 is implicated in poor outcome of paediatric acute lymphoblastic leukemia. Br J Haematol 2013;163: 93–103. 10.1111/bjh.12489 23888996

[8] Wang H, Li M, Zhang R, Wang Y, Zang W, Ma Y, et al, Effect of miR-335 upregulation on the apoptosis and invasion of lung cancer cell A549 and H1299. Tumor Biol 2013;34: 3101–3109. 10.1007/s13277-013-0878-9 23740614

[9] Ronchetti D, Lionetti M, Mosca L, Agnelli L, Andronache A, Fabris S, et al, An integrative genomic approach reveals coordinated expression of intronic miR-335, miR-342, and miR-561 with deregulated host genes in multiple myeloma. BMC Med Genomics 2008;1:37 10.1186/1755-8794-1-37 18700954 PMC2531129

[10] Shi L, Jiang D, Sun G, Wan Y, Zhang S, Zeng Y, et al, miR-335 promotes cell proliferation by directly targeting Rb1 in meningiomas. J Neurooncol 2012; 10.1007/s11060-012-0951-z 22886530

[11] Jiang J, Sun X, Wang W, Jin X, Bo X, Li Z, et al, Tumor microRNA-335 expression is associated with poor prognosis in human glioma. Med Oncol 2012;29: 3472–3477. 10.1007/s12032-012-0259-z 22644918

[12] Wang YX, Zhang XY, Zhang BF, Yang CQ, Chen XM, Gao HJ, Initial study of microRNA expression profiles of colonic cancer without lymph node metastasis. J Dig Dis 2010;11: 50–54. 10.1111/j.1751-2980.2009.00413.x 20132431

[13] Vickers MM, Bar J, Gorn-Hondermann I, Yarum N, Daneshmand M, Hanso JE, et al, Stage-dependent differential expression of microRNAs in colorectal cancer: potential role as markers of metastatic disease. Clin Exp Metastasis 2012;29: 123–132. 10.1007/s10585-011-9435-3 22120473

[14] Shu M, Zheng X, Wu S, Lu H, Leng T, Zhu W, et al, Targeting oncogenic miR-335 inhibits growth and invasion of malignant astrocytoma cells. Molec Cancer 2011;10:59. 1186/1476-4598-10-59. 21592405 10.1186/1476-4598-10-59 PMC3129318

[15] Markou A, Yousef GM, Stathopoulos E, Georgoulias V, Lianidou E, Prognostic significance of metastasis-related microRNAs in early breast cancer patients with a long follow-up. Clin Chem 2014;60: 197–205. 10.1373/clinchem.2013.210542 24132943

[16] Yan Z, Xiong Y, Xu W, Gao J, Chen Y, Wang Z, et al, Identification of has-miR-335 as a prognostic signature in gastric cancer. Plos one 2012;7(7):e40037 10.1371/journal.pone.0040037 22802949 PMC3388997

[17] Xu Y, Zhao F, Wang Z, Song Y, Luo Y, Zhang X, et al, MicroRNA-335 acts as a metastasis suppressor in gastric cancer by targeting Bcl-w and specificity protein 1. Oncogene 2012;31: 1398–1407. 10.1038/onc.2011.340 21822301 PMC3312408

[18] Zucker S, Pei D, Cao J, Lopez-Ortin C, Membrane type-matrix metalloproteinases (MT-MMP). Curr Top Dev Biol 2003;54: 1–74. 12696745 10.1016/s0070-2153(03)54004-2

[19] Osenkowski P, Toth M, Fridman R, Processing, shedding, and endocytosis of membrane type 1-matrix metalloproteinase (MT1-MMP). J Cell Physiol 2004;200: 2–10. 15137052 10.1002/jcp.20064

[20] Kessenbrock IK, Plaks V, Werb Z, Matrix Metalloproteinases: regulators of the tumor microenvironment. Cell 2010;141: 52–67. 10.1016/j.cell.2010.03.015 20371345 PMC2862057

[21] Sato H, Takino T, Coordinate action of membrane-type matrix metalloproteinase-1 (MT1-MMP) and MMP-2 enhances pericellular proteolysis and invasion. Cancer Sci 2010;101: 843–847. 10.1111/j.1349-7006.2010.01498.x 20148894 PMC11158779

[22] Gingras D, Beliveau R, Emerging concepts in the regulation of membrane-type 1 metalloproteinase activity. Biochim Biophys Acta 2010;1803: 142–150. 10.1016/j.bbamcr.2009.04.011 19409422

[23] Strongin AY, Proteolytic and non-proteolytic roles of membrane tyhpe-1 matrix metalloproteinase in malignancy. Biochim Biophys Acta 2010;1803: 133–141. 10.1016/j.bbamcr.2009.04.009 19406172 PMC2823998

[24] Deryugina EI, Quigley JP, Pleiotropoic roles of matrix metalloproteinases in tumor angiogenesis: contrasting, overlapping and compensatory functions. Biochim Biophys Acta 2010;1803: 103–120. 10.1016/j.bbamcr.2009.09.017 19800930 PMC2824055

[25] Rodriguez D, Morrison CJ, Overall CM. (2010) Matrix metalloproteinases: what do they not do? New substrates and biological roles identified by murine models and proteomics. Biochim Biophys Acta 1803: 39–54. 10.1016/j.bbamcr.2009.09.015 19800373

[26] Clark ES, Weaver AM, A new role for cortactin in invadopodia: regulation of protease secretion. Eur J Cell Biol 2008;87: 581–590. 10.1016/j.ejcb.2008.01.008 18342393 PMC2566933

[27] Poincloux R, Lizarraga F, Chavrier P, Matrix invasion by tumour cells: a focus on MT1-MMP trafficking to invadopodia. J Cell Science 2009;122: 3015–3024. 10.1242/jcs.034561 19692588

[28] Hayward SW, Dahiya R, Cunha GR, Bartek J, Deshpande N, Narayan P, Establishment and characterization of an immortalized but non-transformed human prostate epithelial cell line: BPH-1. In Vitro Cell Dev Biol 1995;31A:14–24. 10.1007/BF02631333 7535634

[29] Wang X, Wilson MJ, Slaton JW, Sinha AA, Ewing SL, Pei D, Increased aggressiveness of human prostate PC-3 tumor cells expressing cell surface membrane type-1 matrix metalloproteinase. J Androl 2009;30: 259–274. 10.2164/jandrol.108.006494 19136391

[30] Liu P, Wilson MJ, miR-520c and miR-373 upregulate MMP9 expression by targeting mTOR and SIRT1, and activate the Ras/Raf/MEK/Erk signaling pathway and NF-κB factor in human fibrosarcoma cells. J Cell Physiol 2012;227: 867–876. 10.1002/jcp.22993 21898400 PMC3225649

[31] Sarver AL, Li L, Subramanian S, MicroRNA miR-183 functions as an oncogene by targeting the transcription factor EGR1 and promoting tumor cell migration. Cancer Res. 2010;70: 9570–9580. 10.1158/0008-5472.CAN-10-2074 21118966

[32] Sun L, Yan W, Wang Y, Sun G, Luo H, Zhang J, et al, microRNA-10b induces glioma cell invasion by modulating MMP-14 and uPAR expression via HOXD10. Brain Res 2011;1389:9–18. 10.1016/j.brainres.2011.03.013 21419107

[33] Nakayama I, Shibazaki M, Yashima-Abo A, Mura F, Sugiyama T, Masuda T, et al, Loss of HOXD10 expression induced by upregulation of miR-10b accelerates the migration and invasion of ovarian cancer cells. Int J Oncol 2013;43:63–71. 10.3892/ijo.2013.1935 23670532

[34] Xiao H, Li H, Yu G, Xiao W, Hu J, Tang K, et al, MicroRNA-10b promotes migration and invasion through KLF4 and HOXD10 in human bladder cancer. Oncol Rep 2014;31:1832–1838. 10.3892/or.2014.3048 24573354

[35] Zhang H, Pu J, Qi T, Yang C, Li S, Huang K, et al, MicroRNA-145 inhibits the growth, invasion, metastasis and angiogenesis of neuroblastoma cells through targeting hypoxia-inducible factor 2 alpha. Oncogene 2014;33:387–397. 10.1038/onc.2012.574 23222716

[36] Zhang H, Qi M, Li S, Qi T, Mei H, Huang K, et al, microRNA-9 targets matrix metalloproteinase 14 to inhibit invasion, metastasis, and angiogenesis of neuroblastoma cells. Mol Cancer Ther 2012;11:1454–1466. 10.1158/1535-7163.MCT-12-0001 22564723

[37] Xu M, Wang Y-Z, miR-133a suppresses cell proliferation, migration and invasion in human lung cancer by targeting MMP-14. Oncol Rep 2013;30:1398–1404. 10.3892/or.2013.2548 23783274

[38] Gregoli KD, Jenkins N, Salter R, White S, Newby AC, Johnson JL, MicroRNA-24 regulates macrophage behavior and retards atherosclerosis. Arterioscler Thromb Vasc Biol 2014;34:1990–2000. 10.1161/ATVBAHA.114.304088 24990232

[39] Wang J, Ruan K, miR-335 is involved in the rat epididymal development by targeting the mRNA of RASA1. Biochem Biophys Res Commun 2010;402: 222–227. 10.1016/j.bbrc.2010.10.002 20933506

[40] Lunch J, Fay J, Meehan M, Bryan K, Watters KM, et al, miRNA-335 supresses neuroblastoma cell invasiveness by direct targeting of multiple genes from the non-canonical TGF-β signaling pathway. Carcinogen 2012;33: 976–985. 10.1093/carcin/bgs114 PMC3334516 22382496

[41] Scarola M, Schoeftner S, Schnedier C, Benetti R, miR-335 directly targets Rb1 (pRb/p105) in a proximal connection to p53-dependent stress response. Cancer Res 2010;70: 6925–6933. 10.1158/0008-5472.CAN-10-0141 20713524

[42] Cheng Q, Cao H, Chen Z, Ma Z, Wan X, Peng R, et al, PAX6, a novel target of miR-335, inhibits cell proliferation and invasion in glioma cells. Mol Med Reports 2014; 10.3892/mmr.2014.2150 24737483

[43] Takino T, Guo L, Domoto T, Sato H, MT1-MMP prevents growth inhibition by three dimensional fibronectin matrix. Biochem Biophys Res Comm 2013;436: 503–508. 10.1016/j.bbrc.2013.05.134 23756810

[44] Gonzalo P, Moreno V, Galvez BG, Arroyo AG, MT1-MMP and integrins: ahnd-to-hand in cell communication. Biofactors 2010;36(4):248–254. 10.1002/biof.99 20818710

[45] Ma J, Tang X, Wong P, Jacobs B, Borden EC, Bedogni B, Noncanonical activation of Notch 1 protein by membrane type 1 matrix metalloproteinase (MT1-MM) controls melanoma cell proliferation. J Biol Chem 2014;289: 8442–8449. 10.1074/jbc.M113.516039 24492617 PMC3961669

[46] D’Alessio S, Ferrari G, Cinnante K, Scheerer W, Galloway AC, Roses DF, et al, Tissue inhibitor of metalloproteinase-2 binding to membrane-type 1 matrix metalloproteinase induces MAPK activation and cell growth by a non-proteolytic mechanism. J Biol Chem 2008;283: 87–99. 17991754 10.1074/jbc.M705492200

[47] Strongin AY, Mislocalization and unconventional functions of cellular MMPs in cancer. Cancer Met Rev 2006;25: 87–98. 10.1007/s10555-006-7892-y 16680575

[48] Rozanov DV, Savinov AY, Williams R, Liu K, Golubkov VS, Krajewski S, et al, Molecular signature of MT1-MMP: transactivation of the downstream universal gene network in cancer. Cancer Res 2008;68: 4086–4096. 10.1158/0008-5472.CAN-07-6458 18519667 PMC2659615

[49] Frittoli E, Palamidessi A, Disanza A, Scita G, Secretory and endo/exocytic trafficking ininvadopodia formation: the MT1-MMP paradigm. Eur J Cell Biol 2011;90: 108–114. 10.1016/j.ejcb.2010.04.007 20605060

[50] Willis AL, Sabeh F, Li X-Y, Weiss SJ, Extracellular matrix determinants and the regulation of cancer cell invasion stratagems. J Micros 2013;251:250–260. 10.1111/jmi.12064 PMC6085113 23924043

[51] Chen P, Parks WC, Role of matrix metalloproteinases in epithelial migration. J. Cell Biochem 2009;108: 1233–1243. 10.1002/jcb.22363 19798678 PMC6440477

